# Supplementary material for: Liver biopsies obtained throughout SIV infection reveal evolving interferon stimulated protein expression within distinct monocyte/macrophage subsets
Source: PLoS Pathog. 2025 Sep 26;21(9):e1013175. doi: 10.1371/journal.ppat.1013175 (PMC12543282; doi:10.1371/journal.ppat.1013175)
Supplement: S1 Table — (DOCX) [file ppat.1013175.s001.docx]

**Table S1. Animal information**

| Group | Animal ID | Sex | Age at Study Start (days [years]) | Weight at Necropsy (g) | Necropsy time (weeks post-infection) |
| --- | --- | --- | --- | --- | --- |
| SIV | RM101 | Female | 2311 [6.3] | 3660 | 20* |
| **SIV** | **RM102** | **Male**** | **1730 [4.7]** | **6680** | **32** |
| SIV | RM103 | Female | 1325 [3.6] | 4560 | 20 |
| SIV | RM104 | Female | 3058 [8.4] | 5500 | 12 |
| SIV | RM105 | Female | 2863 [7.8] | 7060 | 32 |
| **SIV** | **RM106** | **Male** | **2892 [7.9]** | **11360** | **32** |
| SIV | RM107 | Female | 2495 [6.8] | 5440 | 32 |
| SIV | RM108 | Female | 2474 [6.9] | 5460 | 27 |
| SIV | RM109 | Female | 2630 [7.2] | 5040 | 9 |
| **Naive** | **RM110** | **Male** | **2070 [5.7]** | **10980** | **32** |
| Naive | RM111 | Female | 1693 [4.6] | 6000 | 32 |
| **Naive** | **RM112** | **Male** | **2064 [5.6]** | **10390** | **32** |
| Naive | RM113 | Female | 2708 [7.4] | 7000 | 32 |
| **Naive** | **RM114** | **Male** | **1778 [4.9]** | **9020** | **32** |
| Naive | RM115 | Female | 2545 [7.0] | 6680 | 32 |
| Naive | RM116 | Female | 2498 [6.8] | 8020 | 32 |
| Naive | RM117 | Female | 1546 [4.2] | 4540 | 32 |

*Animals euthanized before the planned necropsy time point have the week of necropsy shown in red.

**Male animals are shown in bold text.
