# Supplementary material for: Liver biopsies obtained throughout SIV infection reveal evolving interferon stimulated protein expression within distinct monocyte/macrophage subsets
Source: PLoS Pathog. 2025 Sep 26;21(9):e1013175. doi: 10.1371/journal.ppat.1013175 (PMC12543282; doi:10.1371/journal.ppat.1013175)
Supplement: S2 Table — (DOCX) [file ppat.1013175.s002.docx]

**Table S2. Antibodies for immunofluorescence microscopy**

| **Target** | **Clone** | **Host** | **Dilution** | **Source** | **Color** | **Strategy** |
| --- | --- | --- | --- | --- | --- | --- |
| ASS1 | EPR12398 | Rabbit | 1:500 | Abcam | AF488 | Conj. |
| CD68 | EPR20545 | Rabbit | 1:500 | Abcam | AF594 | 1°, 2°, Conj. |
| CD163 | EDHu-1 | Mouse | 1:50 | BioRad | CF405 | In-house conj. |
| CD206 | Polyclonal | Rabbit | 1:750 | Abcam | AF647 | 1°, 2° |
| GS | GT1055 | Mouse | 1:500 | Thermofisher | CF700 | In-house conj. |
| MX1 | CL143 | Mouse | 1:50 | Millipore | AF488  AF594  CF555 | 1°, 2°  1°, 2°  In-house conj. |
