# Supplementary material for: Liver biopsies obtained throughout SIV infection reveal evolving interferon stimulated protein expression within distinct monocyte/macrophage subsets
Source: PLoS Pathog. 2025 Sep 26;21(9):e1013175. doi: 10.1371/journal.ppat.1013175 (PMC12543282; doi:10.1371/journal.ppat.1013175)
Supplement: S3 Table — (DOCX) [file ppat.1013175.s003.docx]

**Table S3. Antibodies and dyes for flow cytometry**

| **Target** | **Clone** | **Host** | **ul/10^6^ cells** | **Source** | **Color** |
| --- | --- | --- | --- | --- | --- |
| CD45 | D05801283 | Mouse | 1.0 | BD Biosciences | BUV737 |
| Live/Dead Yellow | N/A | N/A | 0.3 | Invitrogen | BV570 |
| CD3 | SP34-2 | Mouse | 2.5 | BD Biosciences | APC-Cy7 |
| CD8 | SK1 | Mouse | 2.5 | Biolegend | APC-Cy7 |
| CD20 | 2H7 | Mouse | 2.5 | Biolegend | APC-Cy7 |
| CD11b | ICRF44 | Mouse | 2.5 | Biolegend | BV785 |
| HLA-DR | G46-66 (L243) | Mouse | 2.5 | Biolegend | AF700 |
| CD68 | KP1 | Mouse | 1.0 | Santa Cruz | PerCP-Cy5.5 |
| CD163 | GHI/61 | Mouse | 1.0 | Biolegend | PE |
| CD206 | 19.2 | Mouse | 2.5 | BD Biosciences | APC |
| CCR2 | 48607 | Mouse | 2.5 | BD Biosciences | BV421 |
| Mac387 | MAC387 | Mouse | 0.3 | Abcam | FITC |
| CD14 | M5E2 | Mouse | 1.0 | Biolegend | BV711 |
| CD16 | 3G8 | Mouse | 1.0 | BD Biosciences | PE-Cy7 |
| MX1 | CL143 | Mouse | 1.5 μg | Millipore | CF-633 (in-house conj.) |
