# Supplementary figures and images for: Liver biopsies obtained throughout SIV infection reveal evolving interferon stimulated protein expression within distinct monocyte/macrophage subsets
Source: PLoS Pathog. 2025 Sep 26;21(9):e1013175. doi: 10.1371/journal.ppat.1013175 (PMC12543282; doi:10.1371/journal.ppat.1013175)

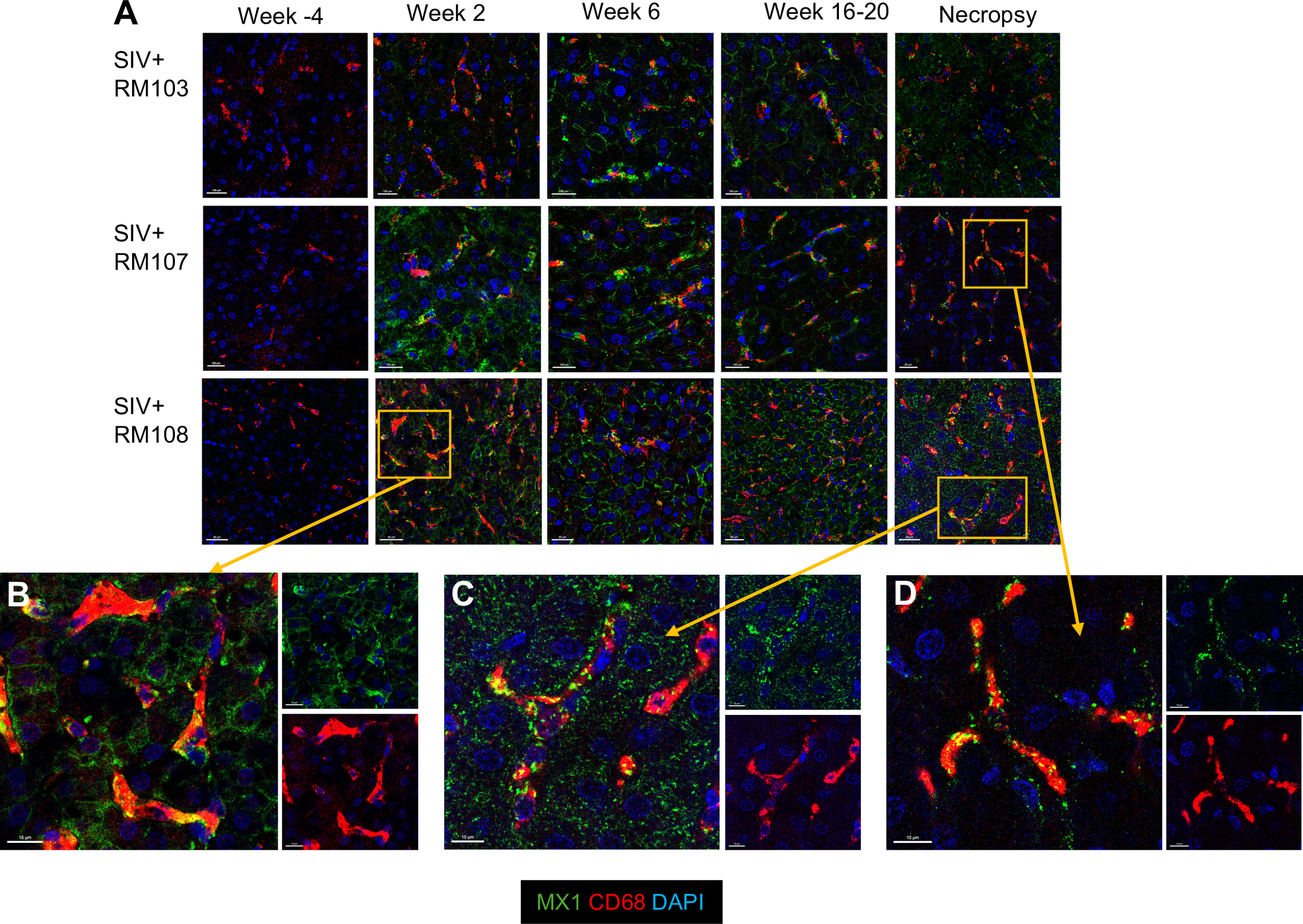

Supplement: S1 Fig — (A) The expression of MX1 and CD68 alongside nuclear staining using DAPI is presented within the livers of three representative SIV-infected macaques. Time points include Weeks –4 (baseline), 2, 4, 16–20 and necropsy. (B) An example image from Week 2 post-infection reveals MX1 expression predominantly within CD68 + cells (monocytes/macrophages), with some signal present in CD68– cells (presumably hepatocytes). (C,D) Two examples of MX1 and CD68 expression at necropsy are shown from two different SIV-infected macaques exhibiting varying degrees of CD68+ and CD68– MX1 staining. (TIF) [file ppat.1013175.s004.tif]

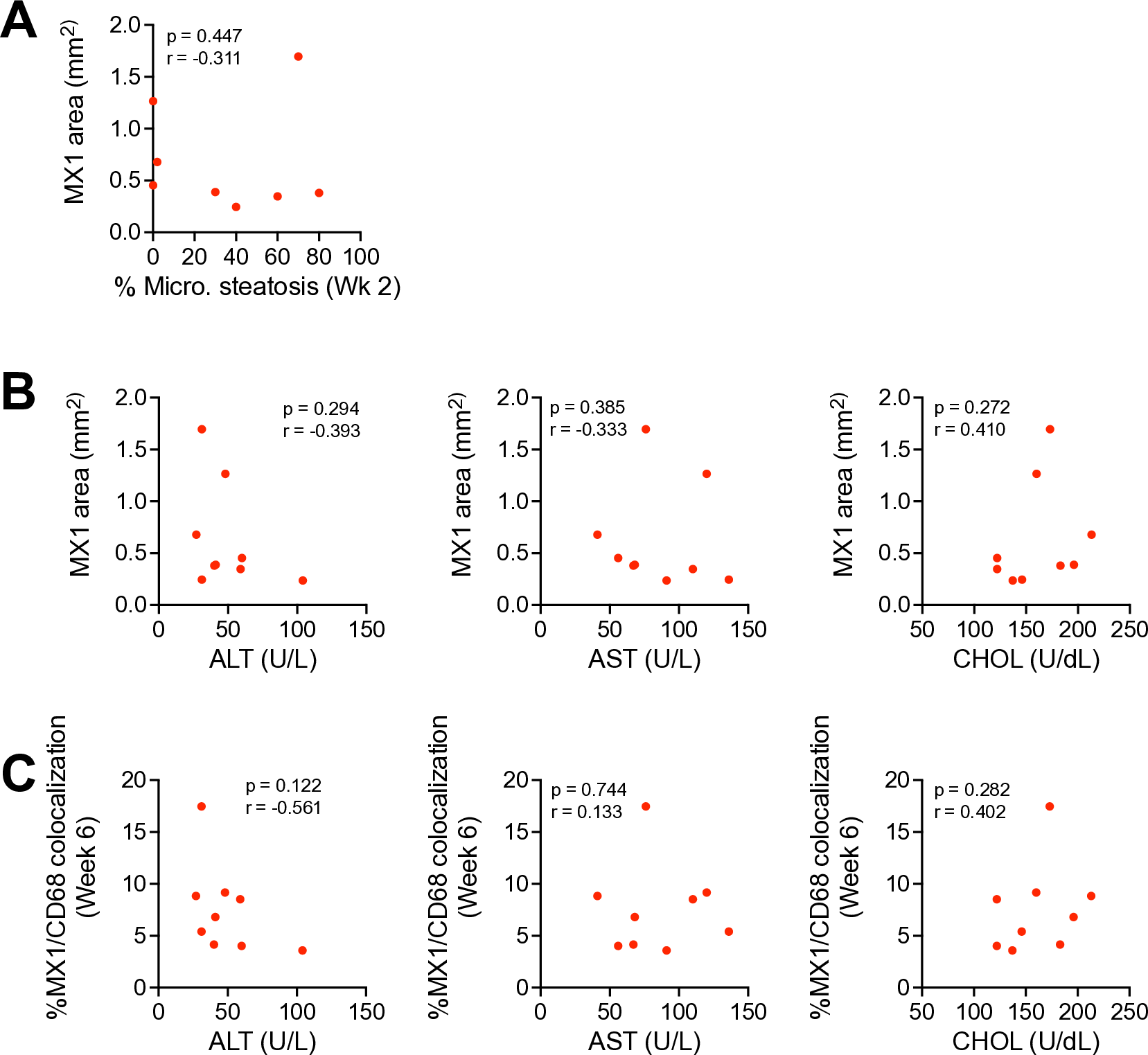

Supplement: S2 Fig — MX1 expression within the livers of the SIV+ macaques at Week 2 measured by immunofluorescence microscopy was plotted against (A) microvesicular steatosis and (B) serum analytes that increased at Week 2 (alanine aminotransferase (ALT), aspartate aminotransferase (AST), and total cholesterol (CHOL)). The proportion of MX1 expression that colocalized with CD68 expression at Week 2 in the SIV+ macaques measured by immunofluorescence microscopy was also plotted against (C) the concentrations of ALT, AST, and CHOL. (TIF) [file ppat.1013175.s005.tif]

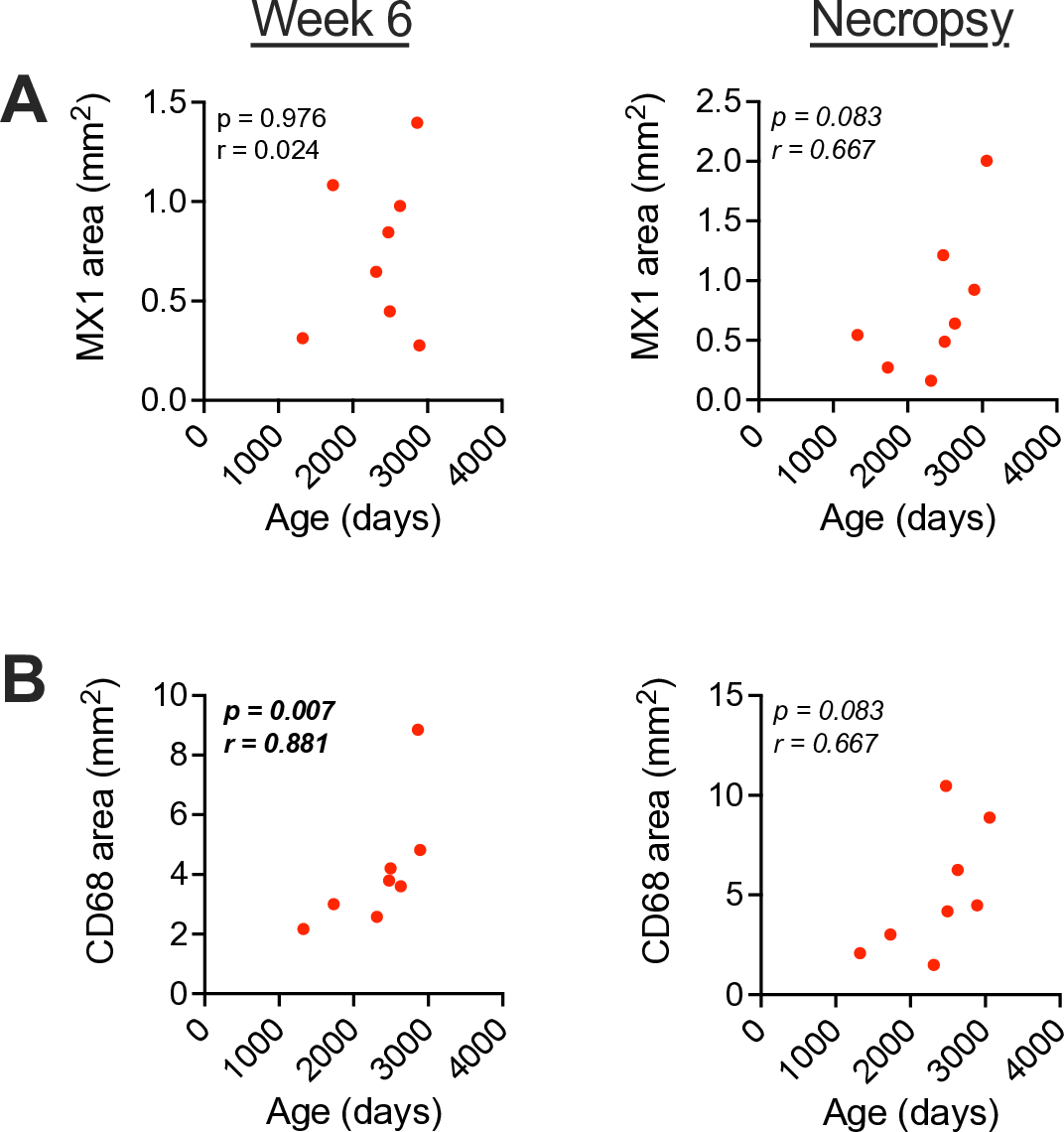

Supplement: S3 Fig — The age of each of the SIV+ macaques enrolled in the study at baseline (measured in days of life) was plotted against the (A) MX1 expression and the (B) CD68 expression within the liver at Week 6 and necropsy time points. (TIF) [file ppat.1013175.s006.tif]

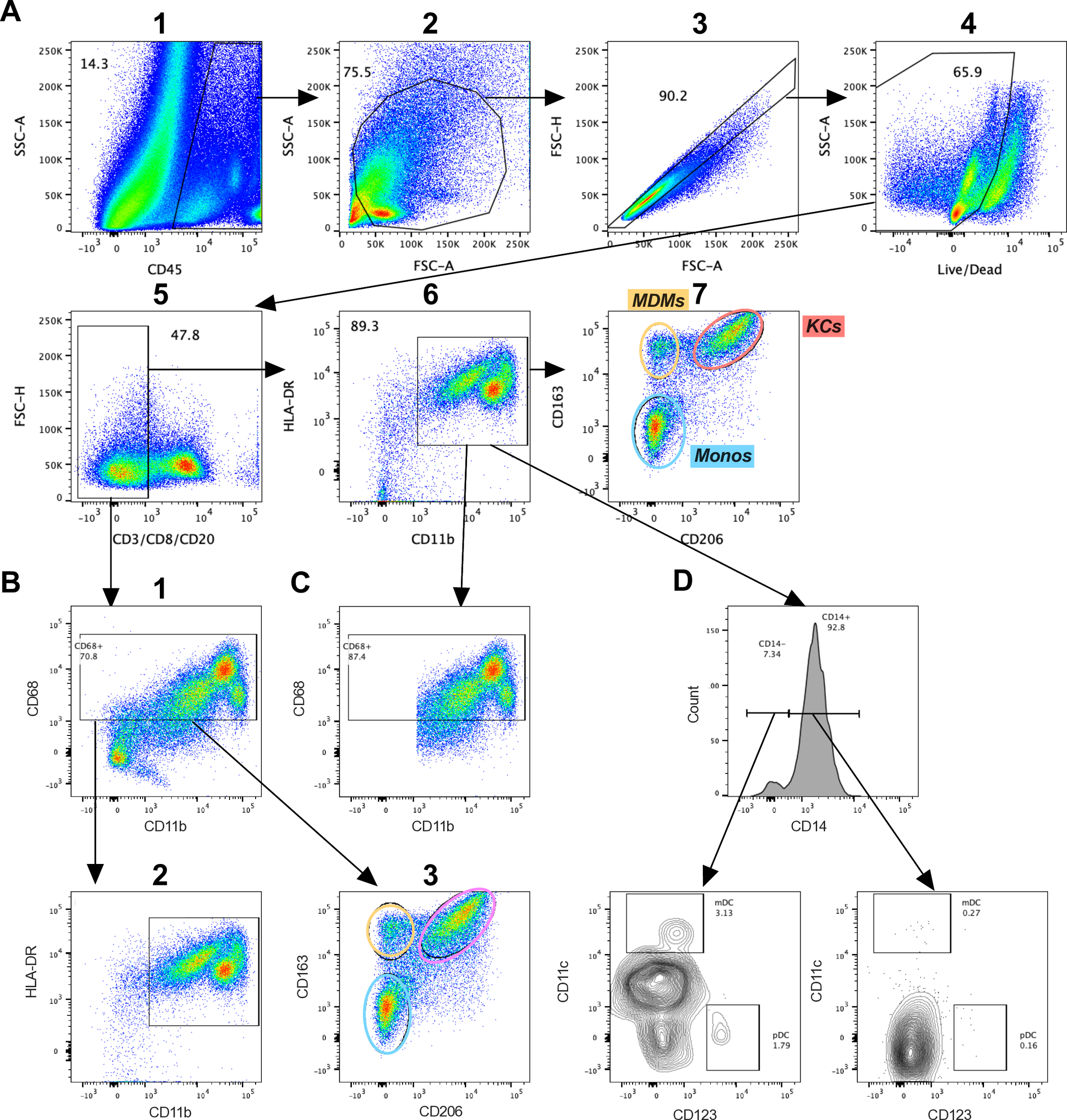

Supplement: S4 Fig — Single cell suspensions of macaque liver were stained with a cocktail of antibodies to identify monocyte/macrophage cell subsets using a gating strategy based on that by Cai, et al [33]. (A) A representative SIV+ macaque at Week 20 is shown. CD45 + cells were first selected [1] and from those, debris was excluded [2] and single cells were selected [3]. Dead cells were excluded [4], and then cells expressing lineage markers CD3, CD8, and CD20 were excluded [5]. Cells were selected that expressed CD11b and HLA-DR with both CD11b-intermediate (CD11bint) and CD11b-high (CD11bhigh) expressing cells captured [6]. CD11b + HLA-DR+ cells were termed “Myeloid cells”, and these were quantified as a proportion of the CD45 + , Non-debris, Single, Live cells, termed “CD45+”. Within the myeloid gate, CD163 + CD206–, CD163 + CD206 + , and CD163– cells were selected and termed monocyte-derived macrophages (MdMs), Kupffer cells (KCs), and monocytes (Monos), respectively [7]. These populations were quantified as a proportion of the CD45 + population defined above. (B) CD3– CD8– CD20– cells were also gated for CD68 expression and the CD68 + cells’ expression of CD11b and HLA-DR as well as of CD163 and CD206 are shown. (C) The expression of CD68 by the CD11b + HLA-DR+ cells from (A) is also shown. (D) Within the CD11b + HLA-DR+ gate, dendritic cells (DCs) were identified by gating CD14 + vs. CD14– cells and divided into CD11c+ myeloid DCs (mDCs) and CD123 + plasmacytoid DCs (pDCs). As expected, the mDC and pDC populations were only present within the CD14– subset, and these cells represented only a small fraction of the myeloid cells in the liver. (TIF) [file ppat.1013175.s007.tif]

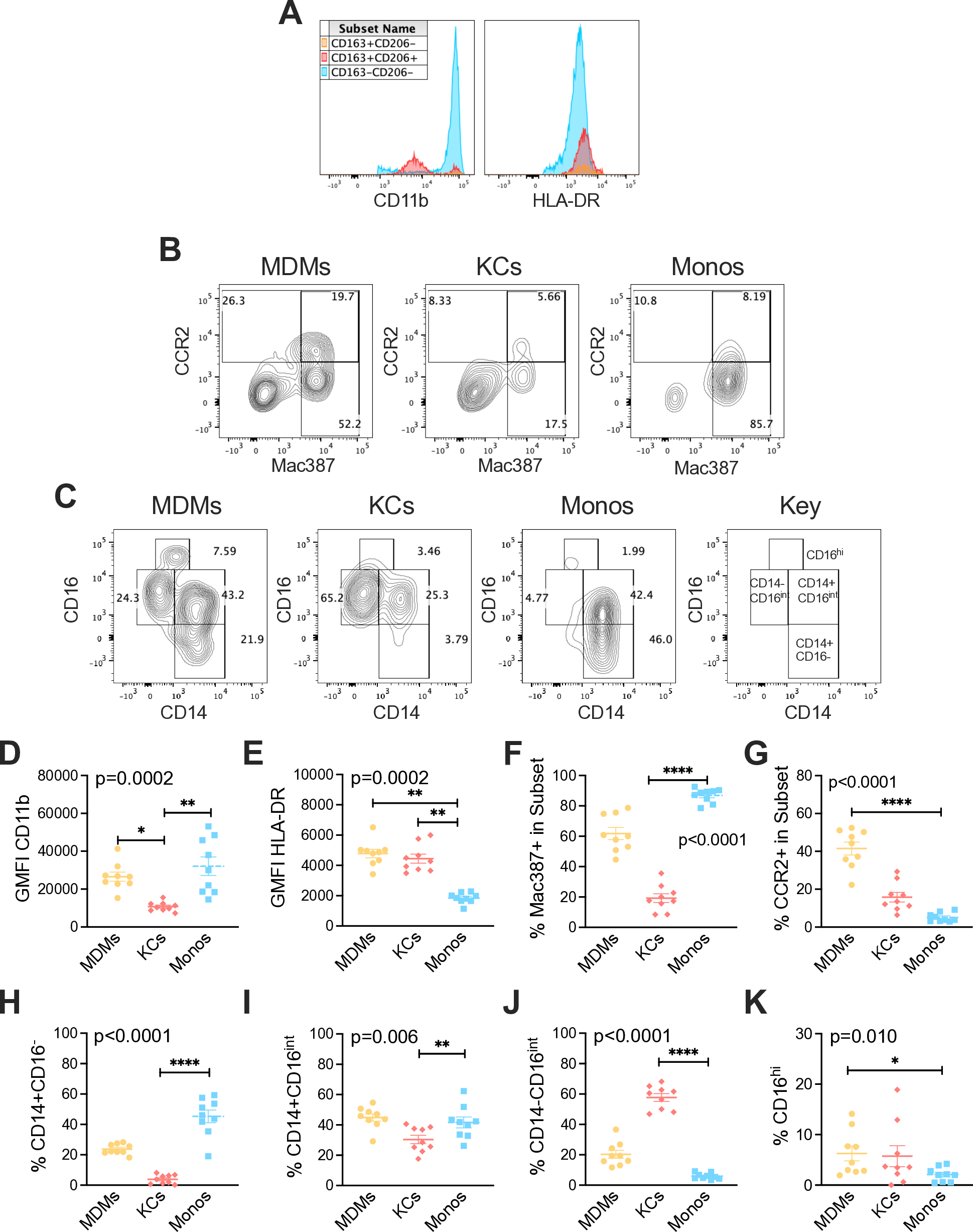

Supplement: S5 Fig — An example of the flow cytometry phenotyping strategy used to define MdM, KC, and Mono signatures is depicted for one macaque at baseline in (A-C) and quantified for all macaques at baseline in (D-K). (A) Histograms displaying the expression intensity (geometric mean fluorescence intensity (GMFI)) of CD11b (left) and HLA-DR (right) within the CD163 + CD206– MdM (yellow), CD163 + CD206 + KC (pink), and CD163–CD206– Mono (aqua) populations. (B) Graphs displaying CCR2-expressing and Mac387-expressing populations within the MdM (left), KC (middle), and Mono (right) subsets of Myeloid cells. (C) Graphs displaying CD16-expressing and CD14-expressing populations within the MdM (left), KC (middle), and Mono (right) populations alongside a key (far right) that distinguishes the myeloid cell subsets based on CD14 and CD16 levels. (D-K) indicate the quantification of the MdM, KC, and Mono populations across all 10 macaques included in this analysis for (D) CD11b GMFI, (E) HLA-DR GMFI, (F) % Mac387 + , (G) % CCR2 + , (H) % CD14 + CD16–, (I) % CD14 + CD16int, (J) CD14–CD16int, (K) CD16high. (TIF) [file ppat.1013175.s008.tif]

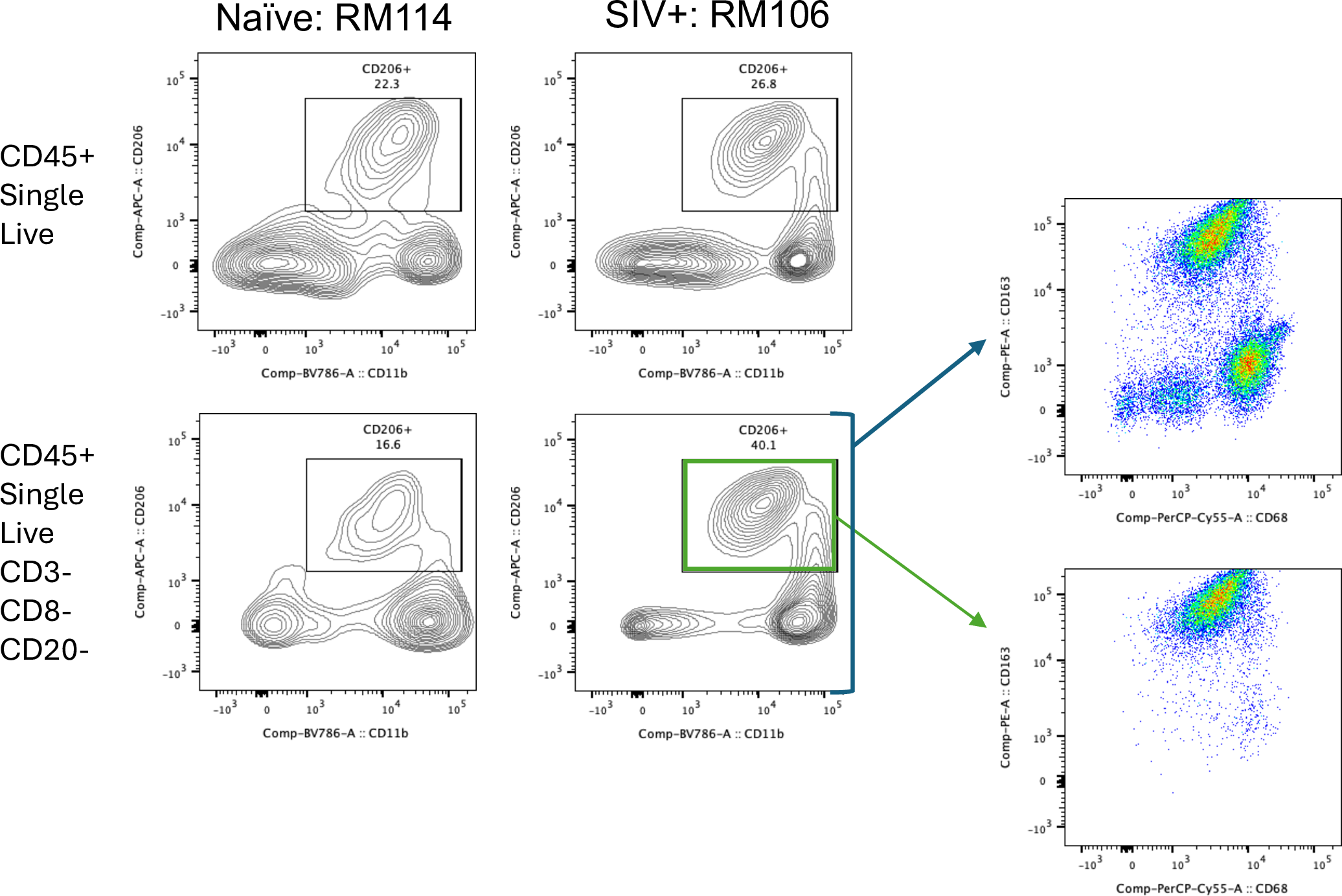

Supplement: S6 Fig — Liver cells from a naïve and an SIV+ macaque at Week 16–20 were gated for CD45+ Single Live cells (top row) or CD45 + Single Live CD3- CD8- CD20- cells (Lineage negative, bottom row) and then examined for the expression of CD206 and CD11b. The Lineage negative population (blue arrow), included CD163 + CD68+ and CD163– CD68++ populations. Gating for only CD206 + cells within the Lineage negative population (green arrow) showed them to be exclusively the CD163 + CD68 + cells. (TIF) [file ppat.1013175.s009.tif]

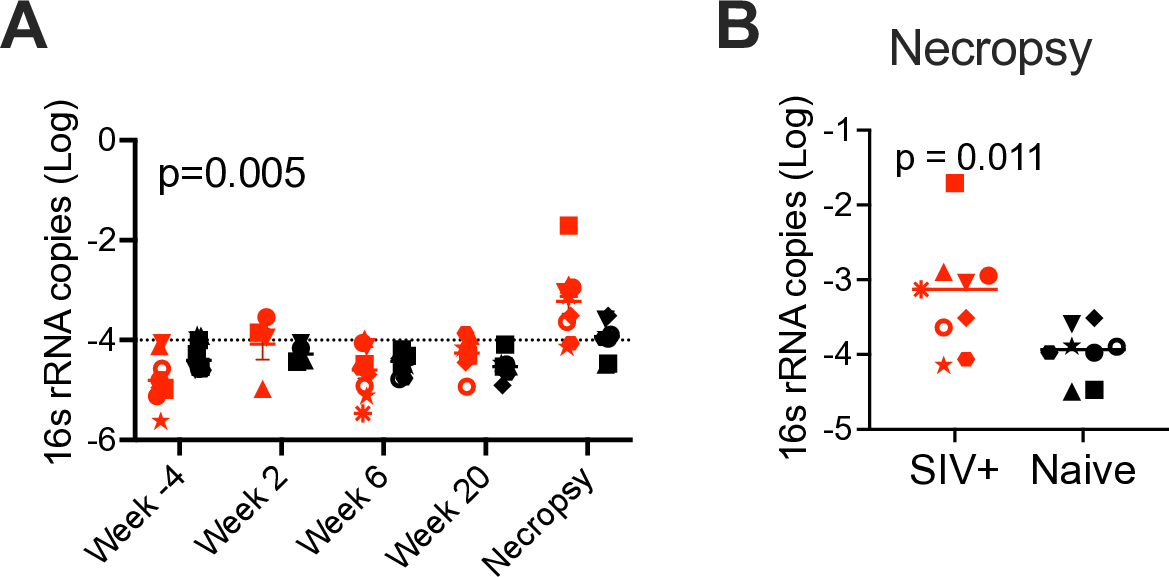

Supplement: S7 Fig — 16s rRNA DNA was measured in total DNA extracted from liver biopsies using the Femto bacterial DNA quantification kit. The number of copies of 16s DNA in liver biopsies from SIV-infected (red) vs. Naïve (black) macaques is shown to differ across the time course (A) and is significant specifically at the necropsy time point (B). (TIF) [file ppat.1013175.s010.tif]
